# Solid Pseudopapillary Neoplasm With Extensive Intratumoral Hemorrhage

**DOI:** 10.7759/cureus.115517

**Published:** 2026-08-31

**Authors:** Hao Ma, Jiaxin Yin, Xianglin Zhang

**Affiliations:** 1 Radiology, The First Affiliated Hospital of Jinzhou Medical University, Jinzhou, CHN; 2 Radiology, The First Affiliated Hospital of Dalian Medical University, Dalian, CHN

**Keywords:** intratumoral hemorrhage, magnetic resonance imaging, pancreatic head, pancreaticoduodenectomy, solid pseudopapillary neoplasm

## Abstract

Solid pseudopapillary neoplasm (SPN) of the pancreas occurs predominantly in young women and commonly contains hemorrhagic and degenerative components that can complicate imaging assessment. We report the case of a 19-year-old woman who presented with abdominal distension and decreased appetite. Multiphasic abdominal CT, including an unenhanced phase, demonstrated a well-circumscribed cystic and solid mass in the pancreatic head, with internally hyperattenuating areas on the unenhanced images and mild heterogeneous enhancement of the non-cystic component. MRI demonstrated peripheral and internal intrinsic T1 hyperintensity, compatible with blood products, markedly heterogeneous T2 signal, and restricted diffusion in a non-cystic portion that also showed post-contrast enhancement, along with an enhancing capsule. The measured apparent diffusion coefficient (ADC) was approximately 0.98 × 10⁻³ mm²/s. Because clotted blood may also restrict diffusion, the diffusion-weighted imaging (DWI) and ADC findings were interpreted together with the conventional and post-contrast images rather than being used alone to identify viable tumor tissue. The patient underwent laparoscopic pancreaticoduodenectomy. Gross examination revealed a cystic and solid tumor containing abundant bloody fluid, and microscopy confirmed conspicuous hemorrhage. Immunohistochemistry showed nuclear and cytoplasmic β-catenin positivity, nuclear progesterone receptor positivity, and CD10 and vimentin positivity, supporting the diagnosis of SPN. Resection margins and sampled lymph nodes were negative. Correlative analysis of CT and MRI findings with pathological and immunophenotypic features may improve diagnostic confidence.

## Introduction

Solid pseudopapillary neoplasm (SPN) accounts for approximately 1-2% of pancreatic tumors and shows a marked predilection for young women. It typically demonstrates an indolent growth pattern and low-grade malignant biological behavior [1-4]. Patients may be asymptomatic or present with nonspecific symptoms such as abdominal distension, abdominal pain, nausea, or early satiety. Because the proportions of solid tissue, cystic degeneration, necrosis, and hemorrhage can vary widely, preoperative diagnosis may be challenging. SPN can arise in any part of the pancreas. Large lesions in the pancreatic head may compress the biliary tract, duodenum, and adjacent vessels and may be mistaken for mucinous cystic neoplasm, pancreatic neuroendocrine tumor, pancreatoblastoma, or pancreatic ductal adenocarcinoma [1,5-7]. In a young woman, however, patient demographics and the combination of a well-defined margin, an enhancing capsule, hemorrhagic components, and gradual mild to moderate enhancement can favor SPN over other pancreatic tumors.

Hemorrhagic SPN may overlap with other cystic and solid pancreatic tumors on imaging, and blood degradation products may also confound diffusion-weighted imaging (DWI) signal, apparent diffusion coefficient (ADC) measurement, and the assessment of true enhancement. Therefore, the key value of this case report lies in the correlation of multiphasic CT and conventional and contrast-enhanced MRI with gross and microscopic pathology, which demonstrates how intratumoral hemorrhage can be recognized and how to avoid simply equating restricted diffusion with viable tumor tissue. This correlation provides a reproducible diagnostic approach for the preoperative interpretation of similar pancreatic masses [1,5,8-10].

## Case presentation

Clinical presentation and initial evaluation

In March 2025, a 19-year-old woman was admitted with abdominal distension and decreased appetite for three months, with worsening symptoms during the preceding four days. Before admission, she had undergone abdominal ultrasonography at a local community health center, which revealed an indeterminate mass in the right upper abdomen, and she was subsequently referred to our hospital for further evaluation and treatment. Her main complaint was upper abdominal fullness. She had no abdominal pain, nausea, vomiting, fever, or jaundice. She had previously been healthy, with no history of smoking, alcohol consumption, chronic disease, infectious disease, trauma, blood transfusion, or drug allergy, and her family history was unremarkable.

At admission, her temperature was 36.3 °C, pulse rate was 92 beats/min, respiratory rate was 19 breaths/min, and blood pressure was 103/75 mmHg. Height was 162 cm, and weight was 43 kg. She was alert and in stable general condition. Abdominal examination showed no rebound tenderness or muscle guarding. Murphy's sign was negative, and no percussion tenderness was present over the liver or either kidney. Shifting dullness was absent, and bowel sounds were three to four per minute.

Imaging examination

Multiphasic abdominal CT, including unenhanced, arterial, venous, and delayed phases, showed a well-circumscribed cystic and solid mass in the pancreatic head measuring approximately 7.0 × 7.3 cm at the largest axial section. On the unenhanced images, the lesion was internally heterogeneous and contained relatively hyperattenuating areas. The non-cystic component showed mild heterogeneous enhancement after contrast administration. The descending duodenum was compressed and displaced, without an infiltrative margin. In view of the patient's age and sex, the circumscribed margin, cystic and solid architecture, internal hemorrhagic appearance, and enhancement pattern, SPN was the leading imaging consideration. Pancreatic pseudocyst and other pancreatic neoplasms were considered less likely (Figure 1).

**Figure 1:**
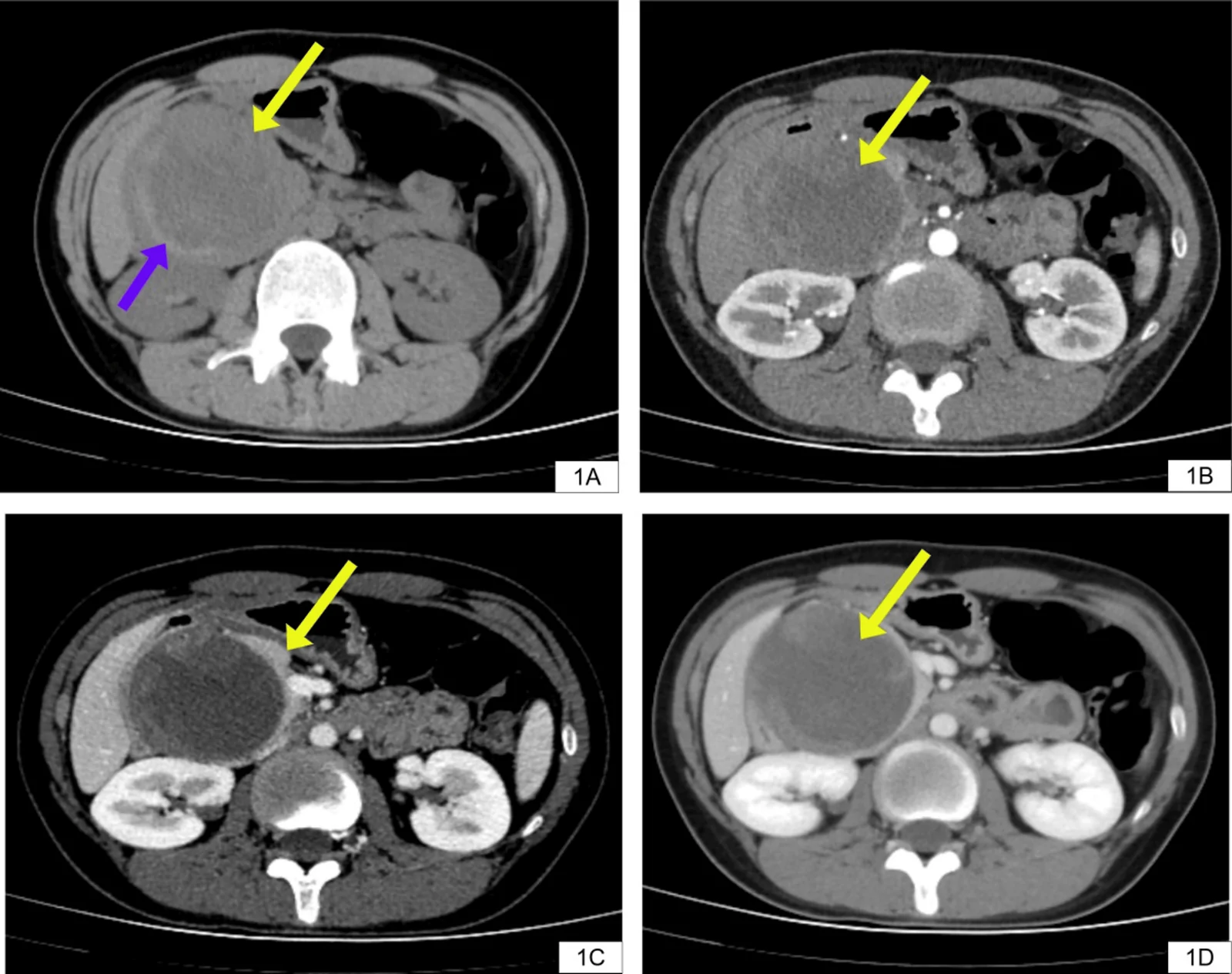
Multiphasic abdominal CT of the pancreatic head mass A: unenhanced phase; B: arterial phase; C: venous phase; D: delayed phase. The lesion is well circumscribed and cystic and solid. The unenhanced image shows internal areas of relatively increased attenuation, while the non-cystic component shows mild heterogeneous enhancement on the post-contrast phases. The descending duodenum is compressed and displaced. The yellow arrow indicates the tumor, while the purple arrow indicates a crescent-shaped, slightly hyperattenuating area, possibly representing intratumoral hemorrhage CT: computed tomography

Magnetic resonance cholangiopancreatography (MRCP) showed an enlarged gallbladder and mild biliary dilatation, with the common bile duct measuring approximately 10 mm in diameter. No definite stenosis was seen at the distal common bile duct, and the main pancreatic duct was clearly visualized (Figure 2). These findings suggested mass effect on the adjacent biliary tract, but patency of the distal common bile duct alone did not exclude a malignant lesion.

**Figure 2:**
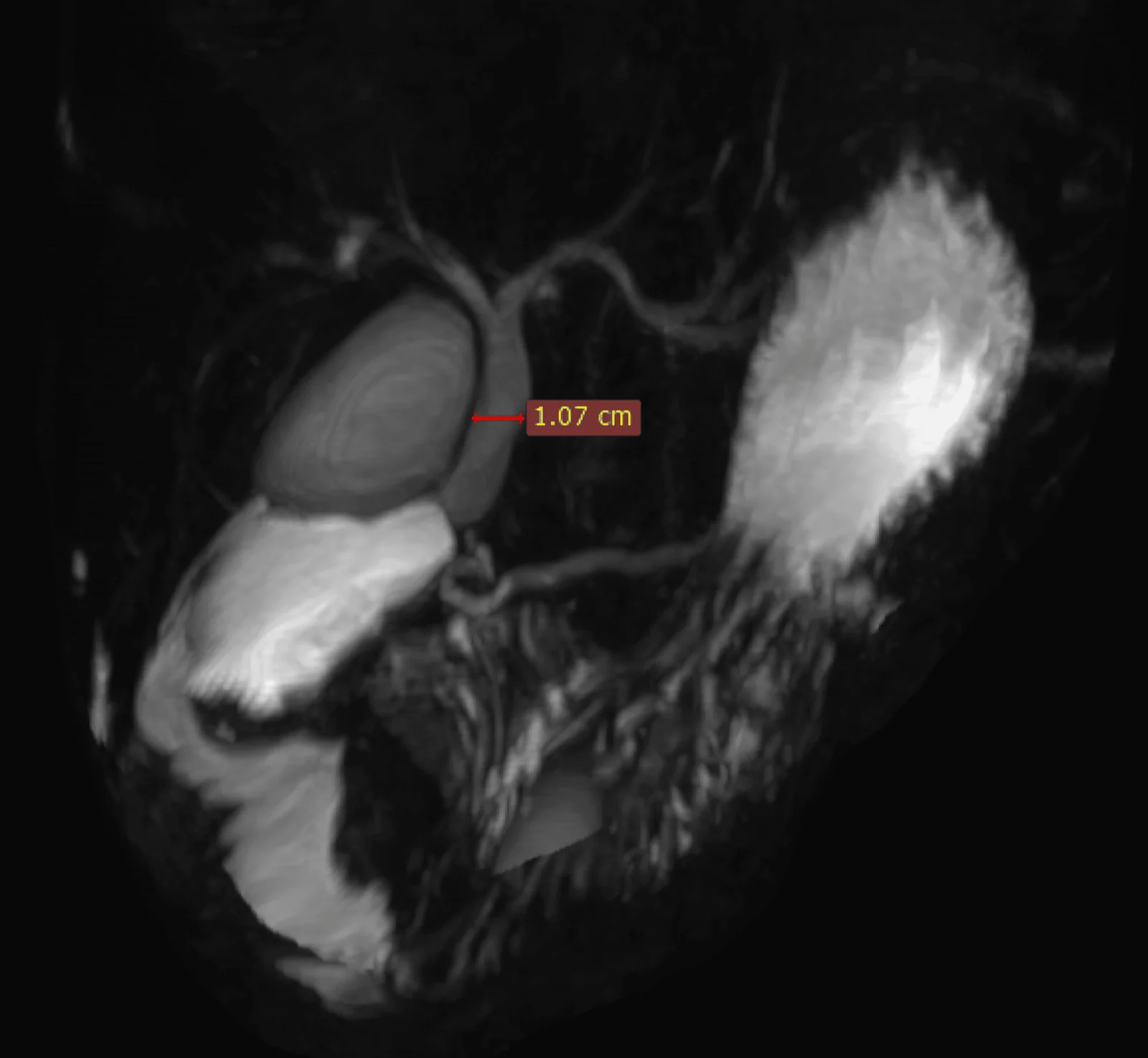
Coronal thick-slab respiratory-triggered 3D MRCP image The image shows an enlarged gallbladder, mild dilatation of the common bile duct (measured at approximately 1.07 cm), a visible distal common bile duct without definite stenosis, and a visible main pancreatic duct MRCP: magnetic resonance cholangiopancreatography (protocol name: VWIP 3D MRCP RT)

Contrast-enhanced pancreatic MRI showed an irregular, approximately round mass in the pancreatic head measuring about 6.2 × 6.5 × 7.6 cm. Peripheral and internal portions of the lesion were intrinsically hyperintense on T1-weighted images, compatible with hemorrhagic products, while T2-weighted images showed markedly heterogeneous high and low signal. DWI demonstrated restricted diffusion in a non-cystic portion of the lesion that also showed post-contrast enhancement, with a measured ADC of approximately 0.98 × 10⁻³ mm²/s. The non-cystic component showed mild to moderate enhancement, and the capsule enhanced. Because blood products may alter DWI signal and ADC measurements, the DWI and ADC findings were interpreted together with the conventional and post-contrast images rather than being used alone to define viable tumor tissue. No obvious enlarged retroperitoneal lymph nodes were seen (Figure 3).

**Figure 3:**
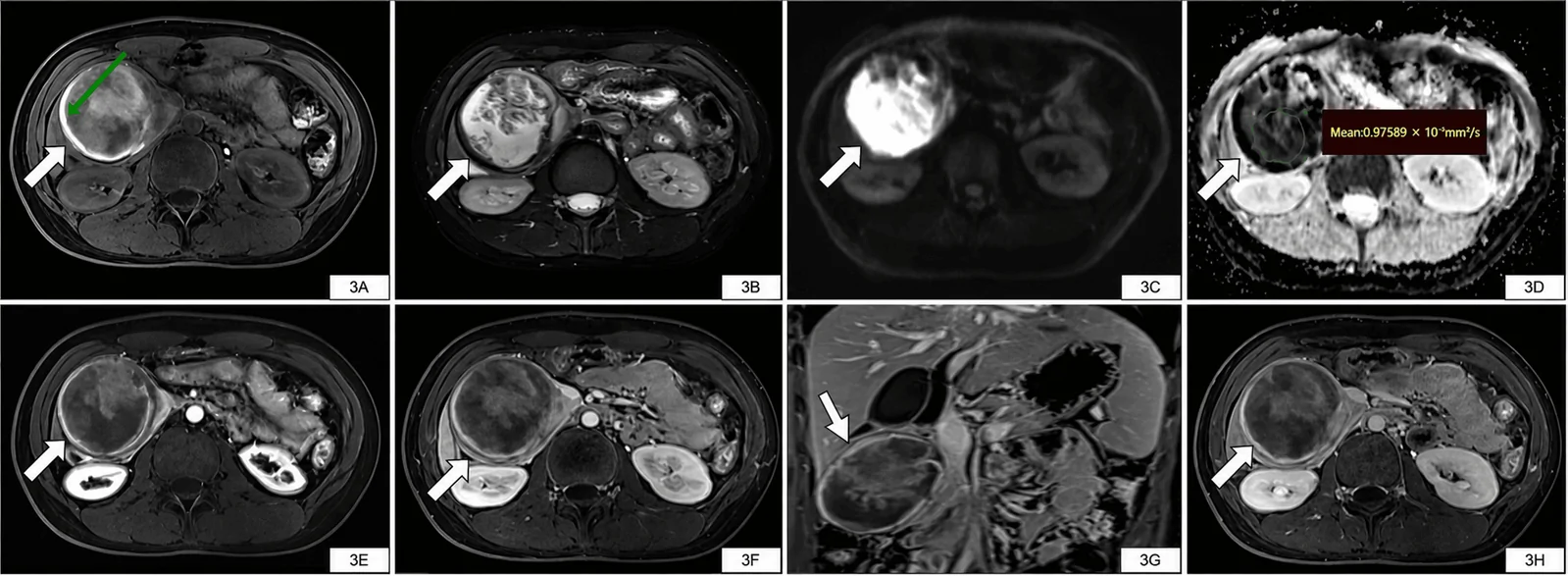
Multiparametric and contrast-enhanced MRI of the pancreatic head mass A: T1-weighted image showing peripheral and internal intrinsic T1 hyperintensity compatible with blood products; B: T2-weighted image showing marked internal heterogeneity; C: DWI; D: ADC map with the measurement ROI; E: arterial phase; F: venous phase; G: coronal venous-phase reconstruction; H: delayed phase. A non-cystic portion shows mild to moderate post-contrast enhancement, and the capsule enhances. Restricted diffusion and the ADC measurement are not specific for viable tumor, because clotted blood products may also restrict diffusion. The white arrow indicates the tumor, while the green arrow indicates a crescent-shaped area of T1 hyperintensity, suggestive of intratumoral hemorrhage, as blood products commonly exhibit high signal intensity on unenhanced T1-weighted images MRI: magnetic resonance imaging; DWI: diffusion-weighted imaging; ADC: apparent diffusion coefficient; ROI: region of interest

MRI was performed on a Siemens MAGNETOM Vida 3.0T scanner using a 60-channel abdominal coil combined with a spine coil. DWI used a SPAIR fat-suppressed single-shot echo-planar imaging sequence with respiratory-triggered acquisition. The b values were 0, 50, 400, and 800 s/mm². TR was approximately 3000 to 5000 ms, TE was no more than 60 ms, slice thickness was 4.0 mm, interslice gap was 0.8 mm, field of view was 360 × 288 mm, matrix was 160 × 128, and the GRAPPA acceleration factor was 2. ADC maps were generated using a monoexponential model. ROI placement was guided by cross-sequence comparison of the conventional and post-contrast images. ROIs were placed in a non-cystic region that showed enhancement, while avoiding obvious T1-hyperintense hemorrhage, necrosis, cystic degeneration, the capsule, and gastrointestinal gas artifacts. Three ROIs measuring approximately 20 to 50 mm² were placed on the largest lesion section and the adjacent sections, and the mean ADC was reported. This sampling strategy reduces but does not eliminate confounding from blood products.

Laboratory and pathological examination

Laboratory testing showed a white blood cell count of 5.68 × 10⁹/L, red blood cell count of 4.32 × 10¹²/L, hemoglobin level of 105 g/L, and platelet count of 610 × 10⁹/L. Coagulation tests, liver and kidney function tests, electrolytes, routine stool examination, and screening for pathogenic microorganisms and infectious diseases showed no significant abnormalities. The postoperative gross specimen showed a hard mass approximately 1.5 cm from the pancreatic transection margin, measuring about 10.0 × 7.0 × 7.0 cm. The cut surface was cystic and solid and contained a large amount of bloody fluid (Figure 4).

**Figure 4:**
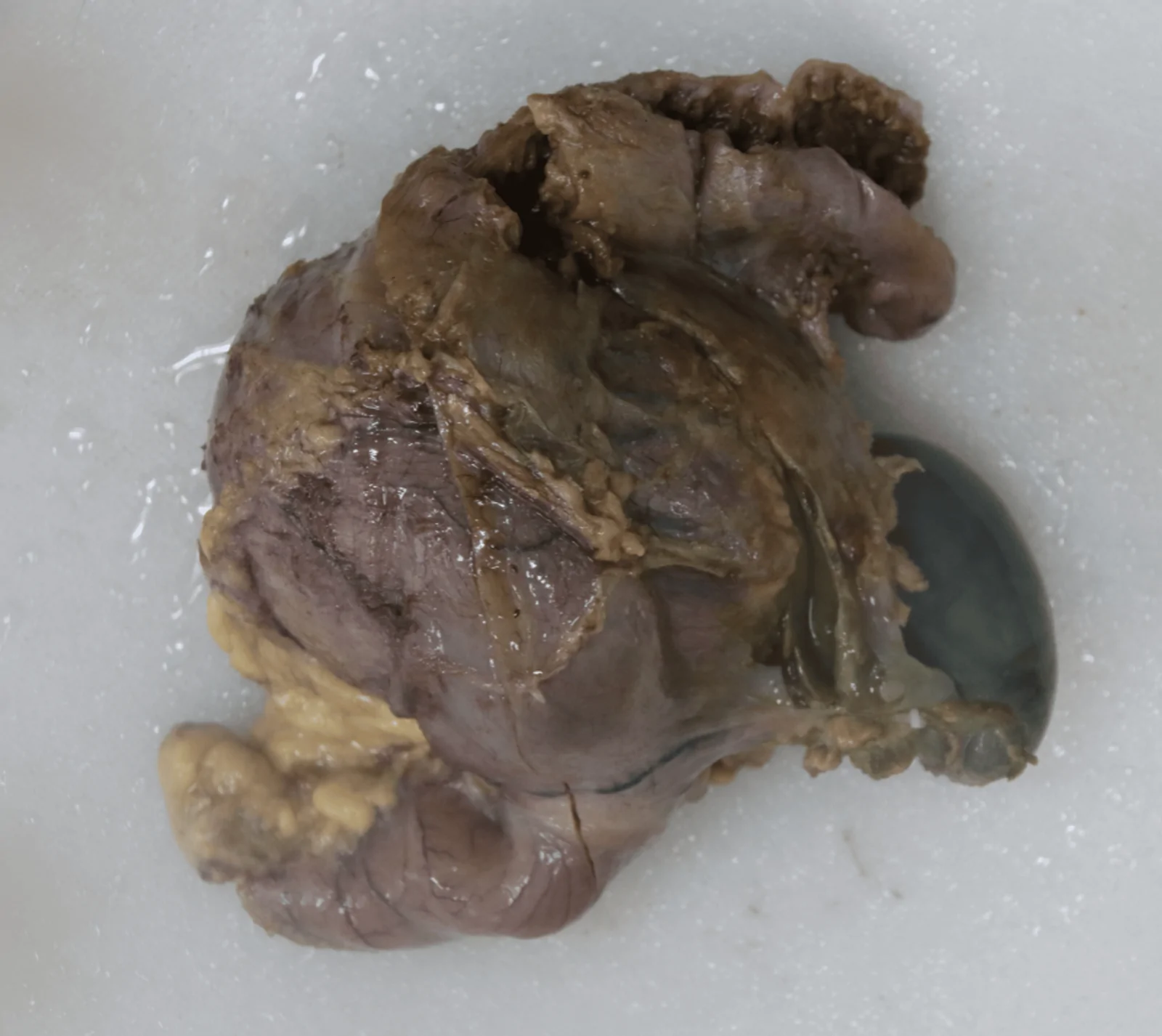
Gross appearance of the surgically resected specimen The pancreatic head mass had a relatively intact capsule, and the cut surface was cystic and solid, and contained a large amount of bloody fluid

Microscopy showed spindle-shaped or round tumor cells with clear or vacuolated cytoplasm; some nuclei were hyperchromatic, and conspicuous hemorrhage was present in the background (Figure 5). Immunohistochemical staining showed nuclear and cytoplasmic positivity for β-catenin, nuclear positivity for progesterone receptor (approximately 70% of cells, with moderate intensity), membranous and cytoplasmic positivity for CD10, and cytoplasmic positivity for vimentin. Synaptophysin showed focal cytoplasmic positivity (approximately 10% of cells), chromogranin A was negative, CK7 was positive in only a small proportion of cells, and the Ki-67 proliferation index was approximately 2%. The pathological diagnosis was SPN. The resection margins of the pancreas, stomach, duodenum, common bile duct, and gallbladder were free of tumor, and no metastasis was identified in the sampled lymph nodes.

**Figure 5:**
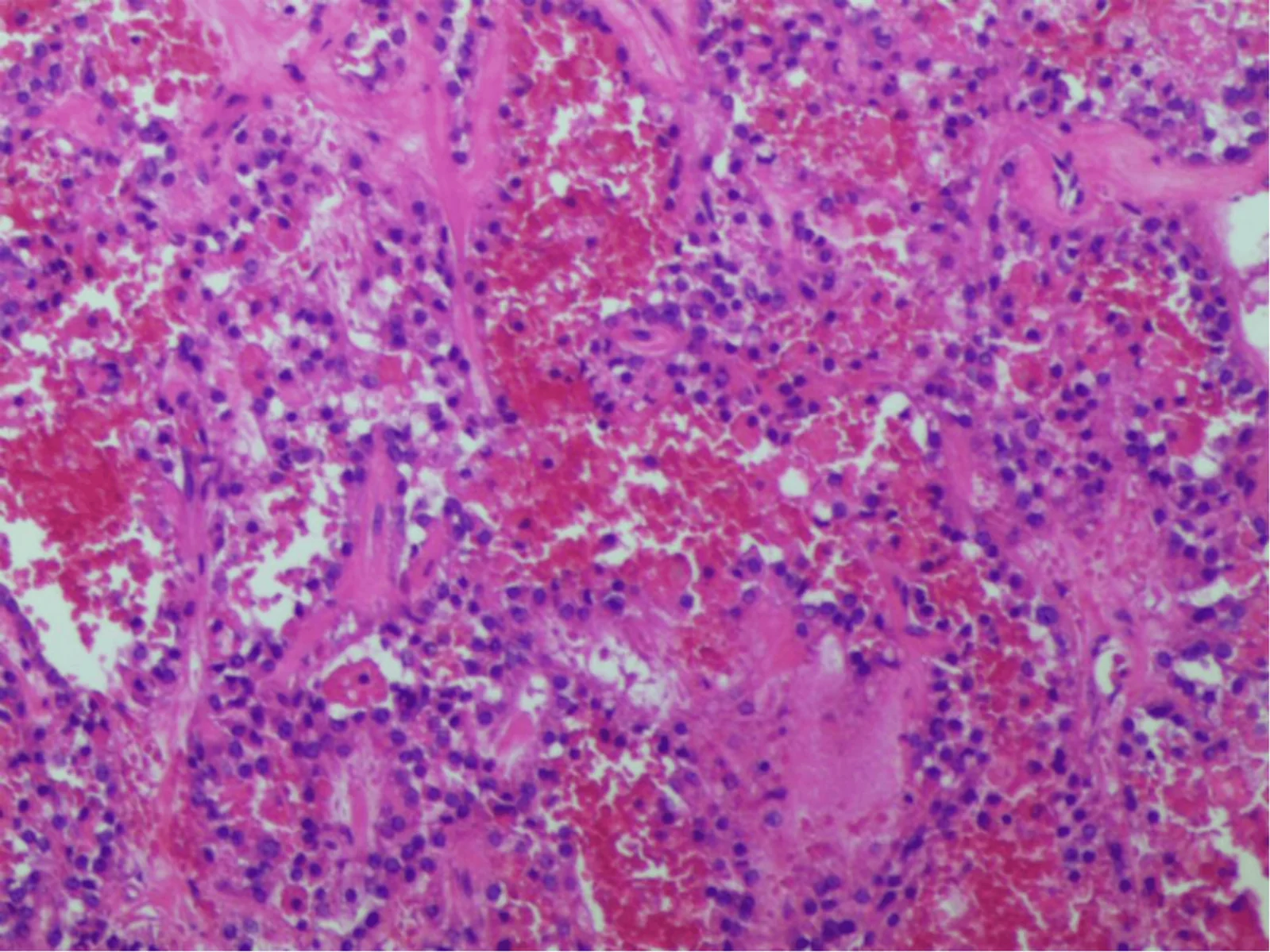
Histological appearance of the tumor The tumor cells were spindle-shaped or round, with clear or vacuolated cytoplasm, and hemorrhage was present in the background. Hematoxylin and eosin staining; original magnification, ×200

Diagnosis, treatment, and follow-up

SPN was the leading preoperative diagnosis proposed by the radiologists, primarily because the patient was a 19-year-old woman and the lesion exhibited several characteristic imaging features, including a well-circumscribed and encapsulated appearance, mixed cystic and solid components, intratumoral hemorrhage, and mild-to-moderate enhancement. The initial surgical team approached this radiological diagnosis with caution, mainly because SPN is relatively rare and therefore less frequently encountered in routine clinical practice. In addition, given the potential consequences of overlooking a malignant pancreatic neoplasm, the surgeons appropriately sought to exclude other malignant or potentially malignant lesions before proceeding with definitive treatment. Therefore, further diagnostic evaluation was performed.

Mucinous cystic neoplasm was considered less likely because it typically occurs in middle-aged women and predominantly involves the pancreatic body or tail. Pancreatic neuroendocrine tumor was also considered less likely because these tumors are generally predominantly solid and characteristically demonstrate more pronounced enhancement during the arterial phase. Pancreatoblastoma was considered unlikely given the patient's age and the absence of typical calcification. A pancreatic pseudocyst was also unlikely because there was no history of pancreatitis or abdominal trauma. Pancreatic ductal adenocarcinoma was considered only as a remote differential diagnosis because it is exceptionally rare in patients of this age, and the lesion lacked an infiltrative margin and typical features of pancreaticobiliary obstruction. Ultimately, postoperative histopathological examination and immunohistochemical analysis established the definitive diagnosis.

On the 10th day after admission, the patient underwent laparoscopic pancreaticoduodenectomy under general anesthesia. Intraoperatively, an approximately 8 × 6 cm cystic and solid mass was identified in the pancreatic head. The capsule was intact, and there was no invasion of the adjacent stomach or colon. Intraoperative frozen sections from the pancreatic transection margin, common bile duct transection margin, and gastroduodenal artery stump showed no tumor involvement; complete postoperative pathological evaluation confirmed an R0 resection.

The patient's postoperative recovery was uneventful, and she resumed her normal daily activities and returned to school after discharge. Whole-abdominal CT performed seven days after discharge showed postoperative changes following pancreaticoduodenectomy and a small amount of pelvic fluid, without other definite abnormalities (Figure 6).

**Figure 6:**
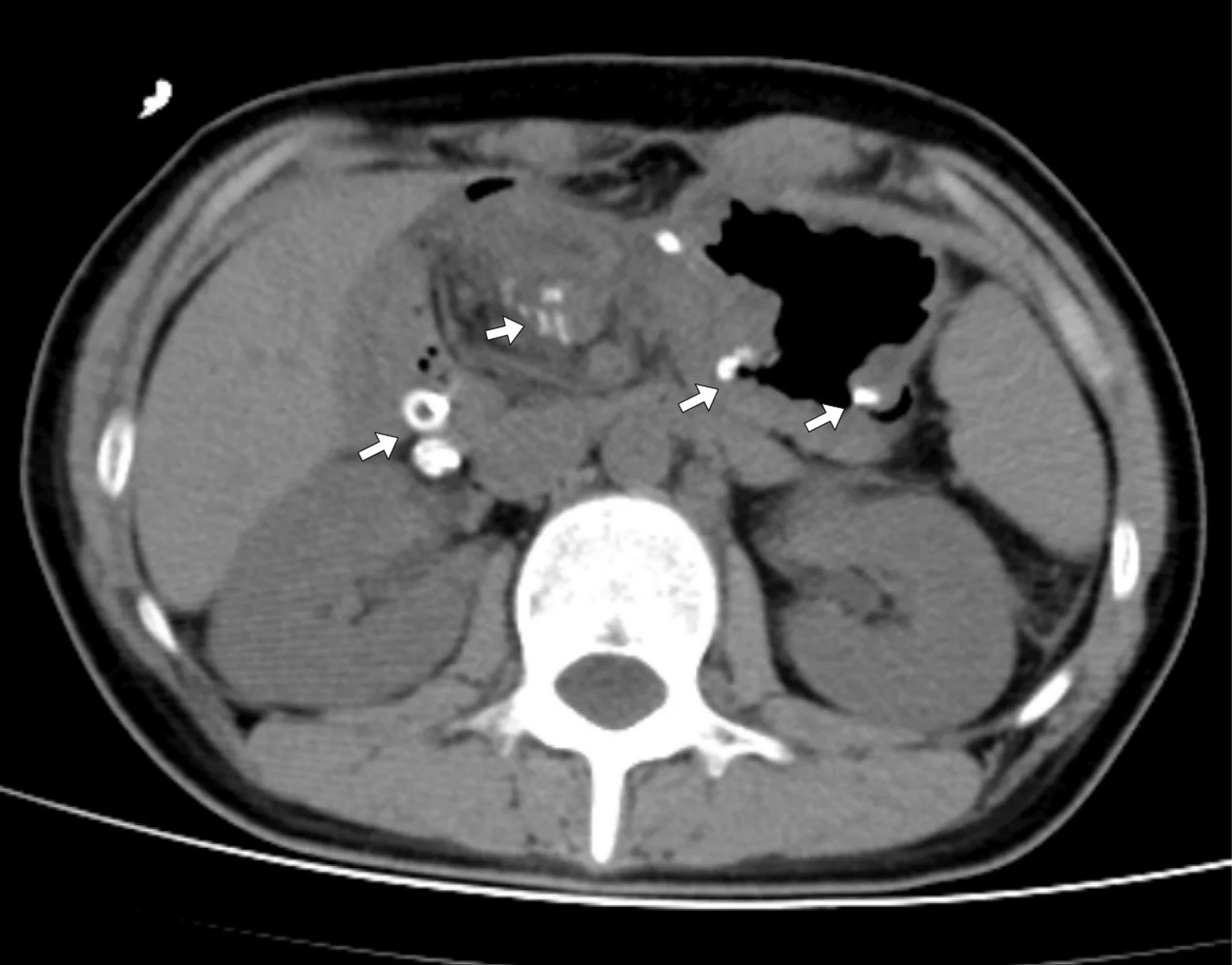
Follow-up CT image of the whole abdomen one week after discharge A follow-up full-abdominal CT scan one week after discharge showed changes after pancreaticoduodenectomy. The drainage tube and surgical stapler (white short arrow) could be seen without other clear abnormalities CT: computed tomography

At 64 days after surgery, unenhanced abdominal CT continued to show postoperative changes, with a smaller amount of pelvic fluid than previously. On the same day, a digital contrast examination of the entire gastrointestinal tract showed that the anastomosis in the operative region was patent and that contrast medium passed smoothly into the small intestine. Six hours later, the contrast medium had reached the colon, with no definite stenosis, filling defect, or delayed gastric emptying (Figure 7).

**Figure 7:**
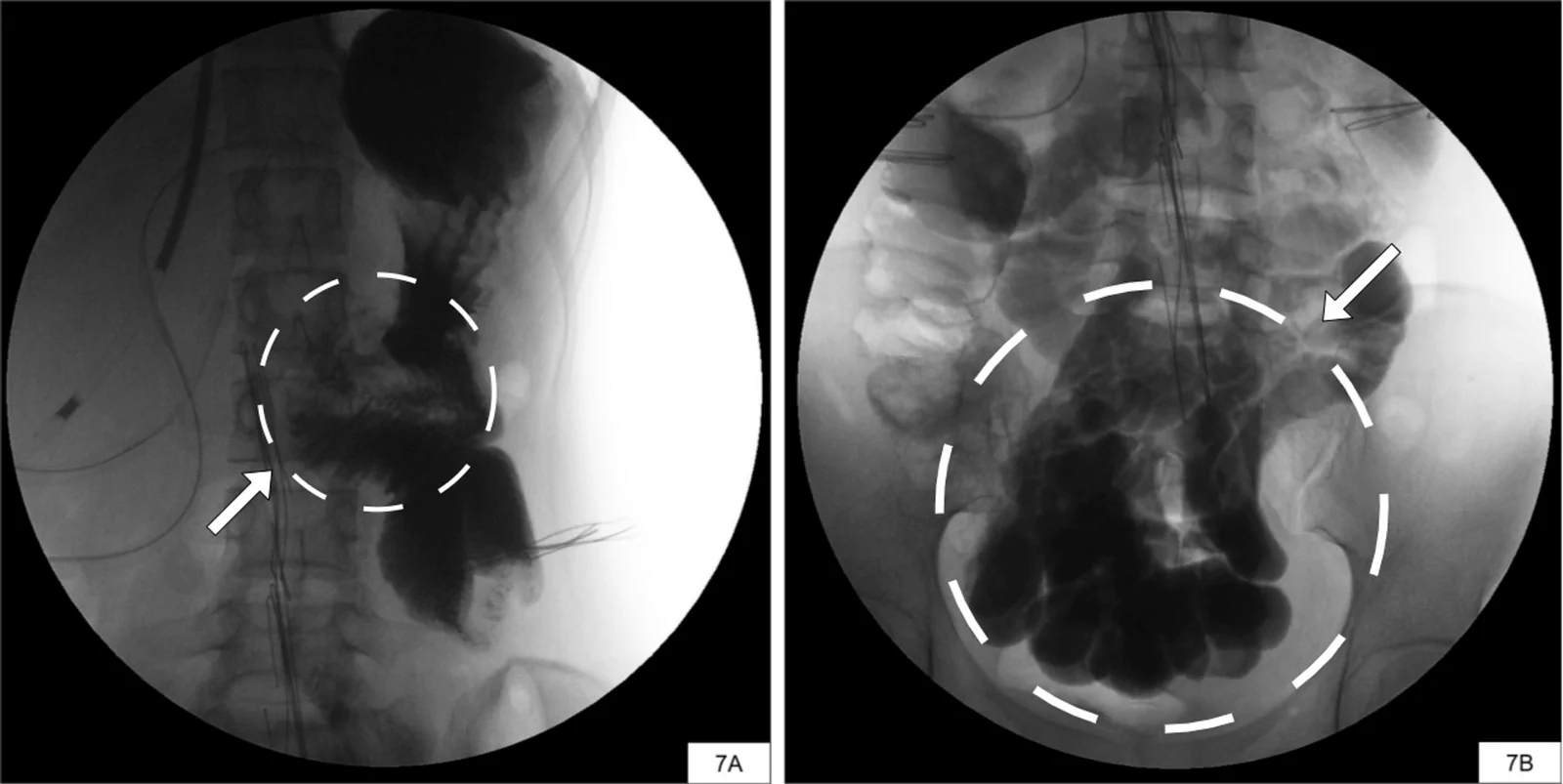
Whole-gastrointestinal-tract digital contrast study after surgery A: After oral administration of contrast medium, the esophagus, stomach, and duodenum were evaluated. Contrast material passed from the gastric antrum into the duodenal bulb, as indicated by the white circle and arrow. B: Six hours later, contrast medium entered the colon with clear visualization. Contrast material within the colon is indicated by the white circle and arrow, demonstrating the characteristic haustral pattern

At the eight-month postoperative telephone follow-up, the patient was attending school in another city. She reported having undergone abdominal ultrasonography and unenhanced abdominal CT at the university hospital; these examinations showed postoperative changes, and the treating clinician informed her that the results were normal. Because our institution did not directly obtain the images or written reports from the university hospital, these findings were recorded based on the patient's telephone account. Her appetite was normal, and her weight had not changed appreciably from before surgery. Although she was experiencing some academic stress, she described her daily life as enjoyable. She planned to return to her home region after graduation, establish an annual follow-up record at our hospital, and undergo regular surveillance examinations.

Patient perspective

"After developing abdominal distension and decreased appetite, I initially thought I had ordinary gastrointestinal discomfort. When the symptoms persisted and worsened, my mother suggested that I visit a clinic. After abdominal ultrasonography at the clinic, I went to the hospital for further evaluation. When I learned that there was a large mass in my pancreas, I felt anxious and worried. I initially thought that I had pancreatic cancer and even considered giving up treatment. During the surgeon's initial consultation and review of the imaging, a rare malignant tumor was considered, which made me feel even more hopeless. Later, a radiology specialist re-reviewed the images and proposed the possibility of a benign lesion, which was good news for me. The doctors then explained the imaging findings, surgical treatment, and pathological results. After laparoscopic pancreaticoduodenectomy, my recovery was generally smooth. Follow-up imaging showed no definite abnormality, and I gradually returned to normal study and daily life. Although I remain concerned about recurrence, I understand that I need to undergo follow-up imaging as recommended by my doctors. This experience showed me that the assessments of different specialties all matter and that accurate interpretation of imaging by radiologists can affect how patients understand their disease and their confidence in treatment."

## Discussion

SPN is an uncommon pancreatic tumor with a marked female predominance and occurs most frequently in adolescents and young adults [1-4,11,12]. Its characteristic molecular alteration is a mutation in exon 3 of the CTNNB1 gene, resulting in aberrant activation of the Wnt/β-catenin signaling pathway, and is closely associated with nuclear accumulation of β-catenin [13,14]. Although molecular testing was not performed in the present case, the histomorphological features and immunophenotype supported the diagnosis of SPN. Most SPNs are slow-growing and have a favorable prognosis after complete surgical resection; nevertheless, local invasion, postoperative recurrence, and distant metastasis have been reported [2-4,11,12,14].

SPN can arise in any part of the pancreas. Its typical imaging appearance is a well-circumscribed cystic and solid mass, usually surrounded by a relatively intact capsule, with marked internal heterogeneity resulting from intratumoral hemorrhage and degenerative changes [1,5,15]. Therefore, the pancreatic-head location in the present case primarily represents an anatomical feature and does not, by itself, explain the extensive intratumoral hemorrhage. The most noteworthy imaging feature in this case was the prominent hemorrhagic phenotype. The relatively hyperattenuating areas on unenhanced CT and the intrinsic T1 hyperintensity within the peripheral and internal portions of the lesion on MRI were concordant with the abundant bloody fluid observed in the gross specimen, and the intratumoral hemorrhage was confirmed histologically. These findings were consistent with the hemorrhagic imaging phenotype of SPN described in previous imaging studies [1,5,15].

The differential diagnosis of SPN should integrate the patient's age and sex with the overall imaging pattern. In a young woman, a capsule, mixed cystic and solid architecture, intratumoral hemorrhage, and the absence of marked arterial-phase hyperenhancement support SPN. Pancreatic neuroendocrine tumors more commonly show arterial-phase hyperenhancement, mucinous cystic neoplasms usually have a different age and location distribution, and pancreatoblastoma occurs mainly in children and may contain calcification [1,5-7]. Pancreatic ductal adenocarcinoma and pancreatic pseudocyst should also be excluded by considering infiltrative margins, pancreaticobiliary duct changes, and a history of pancreatitis or trauma. These features can only narrow the differential diagnosis; definitive diagnosis still depends on histopathological and immunohistochemical examination.

ADC measurement may supplement conventional morphological assessment but should not be used as an independent diagnostic indicator in this case. Through cross-comparison with conventional and contrast-enhanced MRI, an ADC of approximately 0.98 × 10⁻³ mm²/s was measured in a non-cystic, enhancing region of the lesion. Previous studies have shown that blood products at different stages of evolution may demonstrate high DWI signal and low ADC values, while susceptibility effects may also introduce measurement error. Pancreatic ADC measurements are additionally influenced by technical factors such as magnetic field strength, b values, respiratory motion control, sequence parameters, and ROI placement [8,9].

Therefore, the ADC value in this case should be regarded only as a descriptive parameter for an individual patient and not as a diagnostic threshold for SPN, an isolated marker of viable tumor tissue, or a basis for stratifying malignant potential. Because subtraction imaging was not performed, intrinsic T1-hyperintense areas could not always be reliably distinguished from true enhancement. Abdominal MRI subtraction can remove the background signal from intrinsic T1 hyperintensity and more clearly demonstrate true intralesional enhancement [10]. This represents an objective limitation of the imaging assessment in this case.

The typical immunophenotype of SPN includes nuclear β-catenin positivity, frequently accompanied by expression of CD10, vimentin, and progesterone receptor, whereas chromogranin A is usually negative or lacks diffuse expression [13,14]. The immunophenotypic findings in the present case were consistent with these characteristic features. Complete surgical resection remains the preferred treatment for localized SPN. For a large lesion located in the pancreatic head, pancreaticoduodenectomy may be selected according to the relationship of the tumor to the biliary and pancreatic ducts and adjacent critical anatomical structures [1-4,11,12].

The principal strength of this case is the close correlation among multiphasic CT, MRCP, contrast-enhanced MRI, gross pathology, histopathology, and immunophenotype, demonstrating the imaging-pathological features of SPN from multiple perspectives. The patient perspective also indicates that, before pathological confirmation, patients may interpret discussion of several pancreatic tumors as meaning that they already have pancreatic cancer if the distinction between a differential diagnosis and the final diagnosis is not adequately explained. This case therefore suggests that, while undertaking the necessary preoperative differential diagnosis, clinicians should explain diagnostic uncertainty and the purpose of each examination to patients in clear and understandable language.

This case report has several limitations. First, actual follow-up currently extends only to eight months after surgery, and the most recent ultrasonography and unenhanced CT results from the university hospital were reported by the patient by telephone; our institution did not obtain the original images or written reports, and long-term imaging follow-up remains necessary. Second, iron metabolism indices and CTNNB1 mutation status were not assessed, preventing evaluation of the relationships among anemia, molecular features, and prognosis. Third, subtraction imaging was not performed, so intrinsic T1 hyperintensity and true enhancement within the lesion could not be directly and reliably distinguished in all regions. Finally, the patient's hemoglobin level was reduced in the setting of conspicuous intratumoral hemorrhage, but a single case cannot establish a causal relationship between chronic intratumoral hemorrhage and decreased hemoglobin. This hypothesis requires validation in additional cases with complete hematological and iron metabolism assessments.

## Conclusions

In a young woman with a well-circumscribed, encapsulated cystic and solid pancreatic mass, unenhanced CT hyperattenuation and intrinsic T1 hyperintensity should prompt consideration of a hemorrhagic component. DWI and ADC findings should be integrated with morphology and enhancement because clotted blood may also restrict diffusion. Correlation with gross pathology and histology can confirm that the imaging heterogeneity reflects hemorrhage and degeneration, while immunophenotyping establishes the diagnosis of SPN. For resectable localized disease, complete surgical resection can achieve favorable short-term outcomes, but margin assessment and long-term follow-up remain necessary because SPN has low-grade malignant potential.
